# A truncating mutation in EPOR leads to hypo-responsiveness to erythropoietin with normal haemoglobin

**DOI:** 10.1038/s42003-018-0053-3

**Published:** 2018-05-17

**Authors:** Gudjon R. Oskarsson, Ragnar P. Kristjansson, Amy L. Lee, Gardar Sveinbjornsson, Magnus K. Magnusson, Erna V. Ivarsdottir, Stefania Benonisdottir, Asmundur Oddsson, Olafur B. Davidsson, Jona Saemundsdottir, Gisli H. Halldorsson, Joseph Arthur, Gudny A. Arnadottir, Gisli Masson, Brynjar O. Jensson, Hilma Holm, Isleifur Olafsson, Pall T. Onundarson, Daniel F. Gudbjartsson, Gudmundur L. Norddahl, Unnur Thorsteinsdottir, Patrick Sulem, Kari Stefansson

**Affiliations:** 1 deCODE genetics/Amgen, Inc, 101 Reykjavik, Iceland; 2 Faculty of Medicine, University of Iceland, 101 Reykjavík, Iceland; 3 School of Engineering and Natural Sciences, University of Iceland, 101 Reykjavík, Iceland; 4 Department of Clinical Biochemistry, Landspítali University Hospital, 101 Reykjavik, Iceland; 5 Department of Laboratory Hematology, Landspítali University Hospital, 101 Reykjavik, Iceland

## Abstract

The cytokine erythropoietin (EPO), signalling through the EPO receptor (EPO-R), is essential for the formation of red blood cells. We performed a genome-wide association study (GWAS) testing 32.5 million sequence variants for association with serum EPO levels in a set of 4187 individuals. We detect an association between a rare and well imputed stop-gained variant rs370865377[A] (p.Gln82Ter) in *EPOR*, carried by 1 in 550 Icelanders, and increased serum EPO levels (MAF = 0.09%, Effect = 1.47 SD, *P* = 3.3 × 10^−7^). We validated these findings by measuring serum EPO levels in 34 additional pairs of carriers and matched controls and found carriers to have 3.23-fold higher EPO levels than controls (*P* = 1.7 × 10^−6^; *P*_combined_ = 1.6 × 10^−11^). In contrast to previously reported EPOR mutations, p.Gln82Ter does not associate with haemoglobin levels (Effect = −0.045 SD, *P* = 0.32, *N* = 273,160), probably due to a compensatory EPO upregulation in response to EPO-R hypo-responsiveness.

## Abstract (web summary)

Gudjon Oskarsson et al. report the association of a rare variant in the erythropoietin (EPO) receptor gene, *EPOR*, with serum EPO levels in the Icelandic population. The variant leads to a truncation of EPO-R without an effect on hemoglobin levels, indicating a possible feedback mechanism in the generation of red blood cells.

## Introduction

Erythropoietin (EPO) is a cytokine produced and released by the kidney in response to hypoxia^1^. EPO is the primary regulator of erythropoiesis^2^, and exerts its function through the homodimeric EPO receptor (EPO-R). EPO-R is primarily expressed on the surface of erythroid progenitors in bone marrow, but also in a wide variety of tissues including the central nervous system^3–5^. EPO signalling is vital for differentiation, proliferation and survival of erythroid progenitors. EPO-R and EPO homozygous knockout mice die from severe anaemia between embryonic days 13 and 15^4, 6^. Serum EPO levels are regulated via negative feedback loop including an oxygen-sensitive mechanism^7–10^.

Analysis of serum EPO levels is performed in two main clinical contexts; firstly to distinguish between primary and secondary polycythaemias and, secondly, to assess the need for recombinant human EPO (r-HuEPO) replacement therapy, primarily in cases of chronic kidney disease (CKD)^11–13^. C-terminal truncating mutations in *EPOR* leading to a gain of function have previously been reported to cause autosomal dominant primary erythrocytosis, with decreased EPO levels and elevated serum haemoglobin concentration as the main features^14–16^.

A recent genome-wide association study (GWAS) of 6777 healthy subjects in the Netherlands yielded an association between a common single-nucleotide polymorphism (SNP), rs7776054, and serum EPO levels^17^. The variant is located between *HBS1L* and *MYB*, a region containing many common SNPs with associations with haemotological traits^18–26^.

To search for novel associations of sequence variants with EPO levels, we performed a GWAS on Icelanders with serum EPO measurements.

## Results

### GWAS study design

**Fig. 1:**
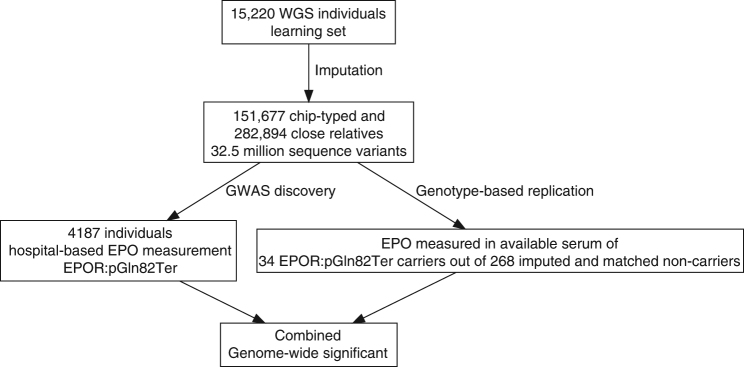
Flowchart depicting the study design. rs370865377[A] (p.Gln82Ter) is the top marker based on corrected *P* value (*P*_Corrected_ = 0.064). Its significance was *P* = 3.3 × 10^−7^ in the GWAS discovery phase. In the replication phase *P* = 1.7 × 10^−6^ and the combined *P* = 1.6 × 10^−11^. WGS whole-genome sequenced

The GWAS discovery phase was performed on 4187 individuals (2% of the Icelandic population) with at least one available EPO measurement (mean number of measurements = 1.4) (Supplementary Table 1). In the GWAS discovery phase, the EPO measurements used were those deemed necessary and performed in a clinical setting at the University Hospital of Iceland between 1994 and 2015. Median value for EPO levels was 13.3 IU L^−1^ (Q1, Q3 quartiles; 8.4 IU L^−1^, 22.7 IU L^−1^). The most common diagnoses observed for this group are presented in Supplementary Table 2. We tested for association between EPO levels and 32.5 million sequence variants (imputation quality (info) > 0.8, minor allele frequency (MAF) > 0.01%) identified through whole-genome sequencing (WGS) of 15,220 Icelanders (~5% of the population) and subsequently imputed into 151,677 chip-typed individuals (~50% of the population of 320,000), as well as 282,894 first- and second-degree relatives of the chip-typed^27^. Of the 4187 individuals with EPO measured, 2994 were chip-typed and 1193 were first or second-degree relatives of the chip-typed (Supplementary Fig. 1). Correlation between genotype and EPO levels was calculated after inverse normal transformation of EPO levels. When testing for association, we used a previously described methodology for weighting genome-wide significance thresholds depending on sequence variant annotation^28^. The significance threshold for loss of function is 2.6 × 10^−7^, for moderate impact is 5.1 × 10^−8^, for low impact is 4.6 × 10^−9^ and is 7.9 × 10^−10^ for all other variants. A flowchart of the study design is presented in Fig. 1.

### GWAS discovery phase

**Fig. 2:**
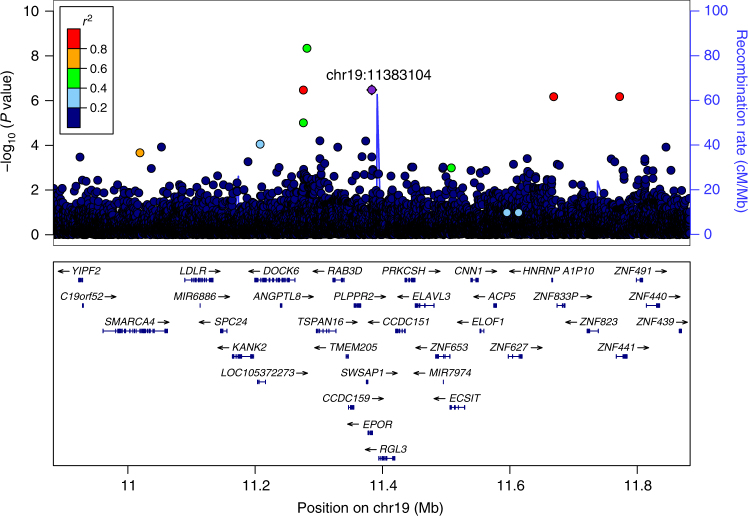
Associations of sequence variants with serum EPO levels at the *EPOR* locus. The mutation p.Gln82Ter in *EPOR* is labelled as a purple diamond, other variants are coloured according to correlation (*r*^2^) with that marker (legend at top-left). –log_10_*P* values are shown along the left *y*-axis and correspond to the variants depicted in the plot. The right *y*-axis shows calculated recombination rates at the chromosomal location, plotted as a solid blue line. No other correlated sequence variants had a more significant corrected *P* value than p.Gln82Ter based on variant annotation; none of the highly correlated variants were coding

The most significant association is with a rare stop-gained variant rs370865377[A] (MAF = 0.09%, p.Gln82Ter, imputation info = 1.00) in *EPOR* that associates with increased serum EPO levels (Effect = 1.47 SD, *P* value = 3.3 × 10^−7^) (Table 1, Fig. 2). We detect no other variants, common or rare, associating significantly with EPO levels. The association of p.Gln82Ter borders on genome-wide significance and the variant is in a biologically relevant gene, the one encoding the EPO receptor (corrected *P* value = 0.064, genome-wide significance threshold for loss-of-function (LOF) mutations = 2.6 × 10^−7^). We do not detect other LOF variants in *EPOR* in our WGS set of 15,220 individuals. Similarly, no quality LOF variants of higher frequency than ours are reported in gnomAD. p.Gln82Ter is located in exon 2 out of 8 exons in *EPOR*. Among the 15,220 whole-genome sequenced individuals are 30 carriers of p.Gln82Ter and imputation of their genotypes into 151,677 chip-typed individuals led to identification of a total of 268 carriers of the mutation. Among those were 7 carriers with serum EPO measurements. The mutation is carried by 1 in 550 Icelanders, whereas it is only detected 6 times in 138,233 genomes reported in gnomAD (MAF = 0.002%, roughly 40× rarer than in Iceland)^29^. The variant is neither present in the HRC (Haplotype Reference Consortium) panel nor the 1000Genomes panel; detecting association to such a rare variant is therefore not achievable using a foreign reference panel.

**Table 1:** *EPOR* sequence variant rs370865377 associating with elevated serum EPO levels in Iceland

								GWAS (*N* = 4187)		Replication (*N* = 34 matched pairs)	Combined
Gene	Chr	Pos (hg38)	rs name	MAF (%)	HGVS	Allele (min/maj)	Info^a^	*P* value^b^	Effect (SD)	*P* value^c^	*P* value^d^
*EPOR*	19	11,383,104	rs370865377	0.09	p.Gln82Ter	A/G	1.00	3.3 × 10^−7^	1.47 (0.91, 2.03)	1.7 × 10^−6^	1.6 × 10^−11^

Results from the initial and replication experiments are presented, along with their combined *P* value. The effect for the initial experiment is 1.47 SD per allele. The carriers in the replication experiment had median EPO levels 3.23-fold higher than their matched controls

*MAF* minor allele frequency, *CI* confidence interval

^a^ Imputation quality score (Info)

^b^ Chi-square test

^c^ Wilcoxon signed-rank test

^d^ Fisher’s combined probability test

### Replication phase

**Fig. 3:**
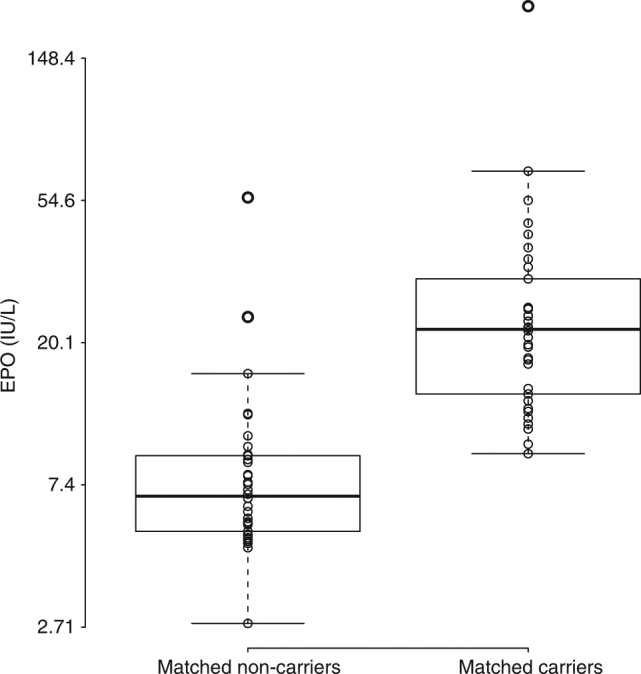
Serum EPO concentration of 34 carriers of rs370865377[A] and 34 non-carriers matched by sex, sample collection date, and age at sampling date. Measurements were performed by ELISA. The bottom and top of each box represent the first and third quartiles, the line inside the box is the median and whiskers represent the ±1.5 times the interquartile range. EPO serum levels are plotted on the log-scaled *y*-axis. The median value of serum EPO concentration of carriers was 3.23-fold higher (median = 22.1 IU L^−1^; Q1 = 14.1; Q3 = 30.0) than that of non-carriers (median = 6.8 IU L^−1^; Q1 = 5.4; Q3 = 9.0) (*P* = 1.7 × 10^−6^)

In order to validate the association between serum EPO levels and p.Gln82Ter, we attempted a replication where we independently measured serum EPO levels in all imputed p.Gln82Ter carriers with available serum sample, as well as in matched controls (*N* = 34 pairs) using enzyme-linked immunosorbent assay (ELISA; Human Erythropoietin Quantikine IVD ELISA kit #DP00; R&D Systems) (Supplementary Table 1). The individuals used in the replication phase were drawn from the overall genotypic dataset as in the GWAS discovery phase, although the individuals in the replication phase had not previously had serum EPO levels measured at the hospital and therefore did not overlap with the individuals used in the EPO GWAS discovery phase. Without knowledge of imputed carriers, a random set of 19,000 Icelanders would have been required in order to identify the same number of carriers (*n* = 34) of p.Gln82Ter. We matched carriers and non-carriers in the replication phase on sex, year of birth, and year of serum sample collection (within 1 year). To estimate the significance of the difference between the two matched groups, we used a Wilcoxon signed-rank test and the average measured EPO value (measured in triplicate). EPO levels were higher in p.Gln82Ter carriers than the matched non-carriers (*P* = 1.71 × 10^−6^, based on Wilcoxon signed-rank test) (Methods, Fig. 3, Supplementary Fig. 2). The median serum EPO levels were 3.23-fold higher in carriers than in the matched non-carriers (median_carriers_ = 22.1 IU L^−1^, median_non-carriers_ = 6.8 IU L^−1^; Supplementary Table 1). The size of the effect detected in the replication phase is expected to represent the effect of the mutation better than the effect detected in the GWAS discovery phase. The association of p.Gln82Ter and EPO levels in the combined GWAS discovery and replication studies reached genome-wide significance (*P* = 1.64 × 10^−11^, based on Fisher’s combined probability test)^30^.

### Transcriptional and translational impact of p.Gln82Ter

**Fig. 4:**
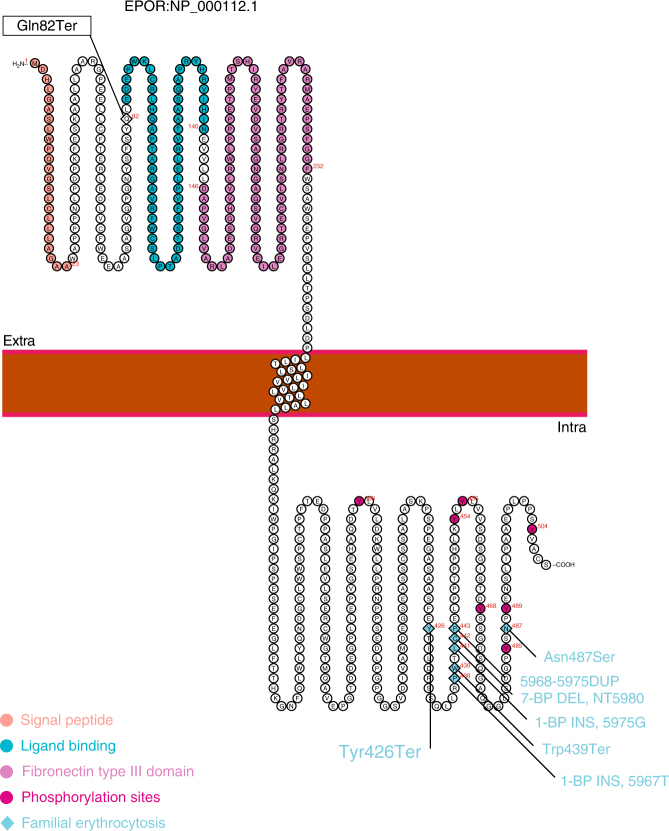
Schematic diagram of the EPO-R protein product (NP_000112.1) depicting relative location of the N-terminal p.Gln82Ter and reported C-terminal truncating mutations. Y454 and Y456 show strongest affinity to negative regulatory agents and are lost in the reported C-terminal truncating mutations^14, 16, 31–34, 39, 52^. Functional regions are represented with colours

The stop-gained mutation at position 82 of EPO-R is predicted to lead to loss of protein function by either nonsense-mediated decay of the mutated messenger RNA (mRNA) or by protein truncation with a resultant fragment consisting of only 81 N-terminal amino acids lacking important functional domains, including the transmembrane domain (Fig. 4)^3^. RNA-sequencing (RNA-seq) of whole blood of 2502 individuals demonstrated similar levels of *EPOR* mRNA in carriers and non-carriers (*P* = 0.48, Effect = 0.30 SD), and allele-specific *EPOR* expression in p.Gln82Ter carriers demonstrated similar mRNA levels of the mutated and wild-type alleles (*P* = 0.46) (6 carriers and 2496 non-carriers; Supplementary Figs. 3 and 4). None of the six carriers used in the RNA analysis had EPO measured in the GWAS discovery phase, but one of them was part of the replication phase.

### Phenome analysis

We tested p.Gln82Ter for association with serum haemoglobin levels in a large set of individuals with haemoglobin measurements (*N* = 273,160, corresponding to 85% of the Icelandic population, geometric mean number of measurements = 1.2). With this sample size, we had 80% power to detect an absolute effect of 0.127 SD (corresponding to 2.03 g L^−1^ or 1.5% of mean) for p.Gln82Ter on haemoglobin levels (Supplementary Fig. 5), but did not detect any association (*P* = 0.32, Effect = −0.044 SD, 95% confidence interval (CI) = −0.13, 0.04) (Table 2). This is in contrast to individuals carrying other reported *EPOR* mutations that associate with decreased EPO levels and elevated haemoglobin levels^14–16, 31–33^. Accordingly, we did not observe association between p.Gln82Ter and stroke, myocardial infarction or venous thromboembolism, phenotypes commonly associated with either elevated haemoglobin in erythrocytosis or adverse effect of r-HuEPO therapy, although we have limited power to detect modest effects due to the low variant frequency (Supplementary Table 3). Furthermore, we did not see any association with lifespan after 50 years of age (*P* = 0.28, Effect = −0.088 SD).

**Table 2:** Association of rs370865377[A] with quantitative phenotypes relevant to blood homoeostasis

Phenotype	*N* measures	*P* value	Effect (SD)	95% CI
Haematocrit	268,689	0.12	−0.068	−0.15, 0.02
Haemoglobin	273,160	0.32	−0.045	−0.13, 0.04
Mean corpuscular volume (MCV)	272,740	0.13	0.080	−0.02, 0.18
RBC count	270,858	0.0093	−0.12	−0.21, −0.03
Platelets	268,587	0.19	0.067	−0.03, 0.17
WBC	273,110	0.95	−0.0030	−0.10, 0.09

We replicated the reported association between the common *HBS1L-MYB* intergenic variant rs7776054[A] and increased serum EPO levels (Supplementary Table 4).

## Discussion

We discovered a rare stop-gained mutation, p.Gln82Ter in EPO-R, present in one out of 550 Icelanders, associating with a threefold increase in EPO levels without an effect on haemoglobin levels.

p.Gln82Ter terminates the EPO-R protein at amino acid 82 out of the 508 amino acid full-length protein, eliminating the intracellular and transmembrane domains^3^. An expected effect would be a reduction in the number of EPO receptors present at the cell surface, leading to EPO-R hypo-responsiveness to EPO. The elevation of EPO seen in carriers of p.Gln82Ter in Iceland is likely a compensation for this hypo-responsiveness which would cause anaemia given normal EPO levels. In contrast, truncating mutations removing only parts of the intracellular EPO-R C-terminus that bind negative regulators have been reported to associate with primary erythrocytosis, with low EPO and high haemoglobin levels (Fig. 4, Supplementary Table 5)^14–16, 32–39^. These mutations make EPO-R hyper-responsive to EPO with a secondary effect of increasing haemoglobin levels, which can be advantageous for athletic performance^16^. These two mutations in EPO-R, the gain of function with increased haematocrit and low levels of EPO and the loss of function with normal haematocrit and high levels of EPO, demonstrate that the feedback mechanisms in the generation of red blood cells appear to be more sensitive to the need to provide sufficient oxygen carrying capacity than they are to the deleterious effects of high haematocrit.

r-HuEPO is used in the treatment of anaemia in CKD and malignancies^40, 41^. If requiring treatment, carriers of p.Gln82Ter may require higher levels of r-HuEPO. The administration of higher r-HuEPO has been found to increase mortality in CKD^42^, but an understanding of what will happen to p.Gln82Ter carriers compared to non-carriers requires further examination.

## Methods

### Study subjects

Erythropoietin measurements of 4187 Icelanders were obtained from The National University Hospital of Iceland from 1994 to 2015. Of these, 2994 were chip-typed using Illumina chips and their genotypes were imputed using long-range phased haplotypes. Genotype probabilities were computed for 1,193 individuals with chip-typed first or second-degree relatives (Fig. 1).

All participating individuals who donated blood, or their guardians, provided written informed consent. The family history of participants donating blood was incorporated into the study by including the phenotypes of first- and second-degree relatives and integrating over their possible genotypes.

All sample identifiers were encrypted in accordance with the regulations of the Icelandic Data Protection Authority. Approval for the study was provided by the National Bioethics Committee (ref: VSNb2015010033-03.12).

### Whole-genome sequencing and Illumina chip genotyping

Genotypes for individuals in both the GWAS discovery and replication phases were obtained from a large set created by whole-genome sequencing 15,220 Icelanders participating in various disease projects at deCODE genetics, sequenced to an average genome-wide coverage of 34×. Sequencing was performed using the following three different library preparation methods and sequencing instruments from Illumina: (i) the standard TruSeq DNA library preparation method; Illumina GAIIx and/or HiSeq 2000 sequencers; (ii) the TruSeq DNA PCR-free library preparation method; Illumina HiSeq 2500 sequencers; and (iii) the TruSeq Nano DNA library preparation method; Illumina HiSeq X sequencers. SNPs and indels in the whole-genome sequencing data were identified using the Genome Analysis Toolkit (GATK) HaplotypeCaller, subject to filters based on GATK best practices^43^. Genotype calls were improved by using information about haplotype sharing, taking advantage of the fact that all the sequenced individuals had also been chip-typed and long-range phased. The effects of sequence variants on protein-coding genes were annotated using the Variant Effect Predictor (VEP) using protein-coding transcripts from RefSeq. In addition, these variants have been imputed into 151,677 Icelanders (around 50% of the population) who have been genotyped using Illumina SNP chips (Supplementary Table 6). Of imputed variants with a MAF over 0.1%, 96.7% were imputed with information over 0.8, and only variants with imputation information over 0.8 were tested in the current study. Genotype probabilities for untyped relatives of chip-typed individuals was also calculated based on Icelandic genealogy (Fig. 1). The process used for whole-genome sequence sequencing of Icelanders, and the subsequent imputation from which the data for this analysis were generated, has been extensively described in recent publications^44, 45^.

### Association analysis

We tested 32,554,515 variants (with imputation information > 0.8 and MAF > 0.01%) identified from the whole-genome sequencing of 15,220 Icelanders (5% of the population) for association with EPO serum levels^45^.

Serum EPO measurements were corrected for sex and year of birth. The data were inverse normal transformed to have a standard normal distribution. Generalised linear regression models were used to test for associations between sequence variants and quantitative traits, assuming an additive genetic model. Let *y* be the vector of quantitative measurements, and let *g* be the vector of expected allele counts for the SNP being tested. We assume the quantitative measurements follow a normal distribution with a mean that depends linearly on the expected allele at the variant and a variance covariance matrix proportional to the kinship matrix:

$$y\sim {\cal N}\left( {\alpha + \beta g,2\sigma ^2{\mathrm{\Phi }}} \right),$$

where

$${\mathrm{\Phi }}_{ij} = \left\{ {\begin{array}{*{20}{c}} {\frac{1}{2},i = j} \\ {2k_{ij},i \ne j} \end{array}} \right.$$

is the kinship matrix as estimated from the Icelandic genealogical database. Logistic regression was used to test for association between sequence variants and binary traits. Other available individual characteristics that correlate with disease status were also included in the model as nuisance variables. These characteristics were: sex, county of birth, current age or age at death (first- and second-order terms included), blood sample availability for the individual and an indicator function for the overlap of the lifetime of the individual with the timespan of phenotype collection. Testing was performed using the likelihood ratio statistic.

We used linkage disequilibrium (LD) score regression to account for distribution inflation in the dataset due to cryptic relatedness and population stratification^46^. Using a set of about 1.1 million sequence variants with available LD score, we regressed the χ^2^ statistics from our GWAS scan against LD score and used the intercept as correction factor. The correction factor for serum EPO level was estimated to be 0.98. Since we observed a slight distribution deflation (0.98) we did not use the correction factor to increase significance.

In the replication study, the data were not normally distributed and thus we performed Wilcoxon signed-rank test to estimate significance. We also performed paired Student's *T* test for log-transformed serum EPO values (*P* = 4.9 × 10^−8^), yielding similar results as the Wilcoxon signed-rank test.

### Significance thresholds

We applied genome-wide significance thresholds corrected for multiple testing using adjusted Bonferroni procedure weighted for variant classes and predicted functional impact. With 32,463,443 sequence variants being tested, the weights given in Sveinbjornsson et al.^28^ were rescaled to control the family-wise error rate. The adjusted significance thresholds are 2.6 × 10^−7^ for variants with high impact (*N* = 8464), 5.1 × 10^−8^ for variants with moderate impact (*N* = 149,983), 4.6 × 10^−9^ for low-impact variants (*N* = 2,283,889), 2.3 × 10^−9^ for other variants in Dnase I hypersensitivity sites (*N* = 3,913,058) and 7.9 × 10^−10^ for all other variants (*N* = 26,108,038).

### RNA-sequencing analysis

In total, whole blood from 2502 individuals were RNA-sequenced. The preparation of poly(A)^+^cDNA sequencing libraries and RNA-seq were carried out as described before^47^. Majority of the samples (*N* = 2074) were sequenced with read length 2 × 125, and in some instances read lengths 2 × 101 (*N* = 220) or 2 × 76 (*N* = 208). Reads were aligned to GRCh38 using TopHat version 2.0.12 with a supplied set of known transcripts in GTF format (RefSeq hg38)^48^.

RNA libraries were excluded if the number of mapped reads were less than 10^7^ or number of mapped read pairs were less than 10^6^ or if the mapping rate of the first or second read-end fell below 80% relative to the mapping of the other read-end. Genotype concordance was determined by comparing imputed genotypes to those derived from RNA-seq. Samples surpassing exclusion had median 106 million mapped reads (90–123 M (Q1–Q3)).

HTSeq-count was used to count fragments aligning to genes^49^. Count values were normalised with the Trimmed Mean of M-values method implemented within edgeR (v. 3.12.1) of the Bioconductor package^50^. Generalised linear regression assuming additive genetic effect as described before^44^ was performed on rank-transformed RNA expression estimates from whole blood (*N* = 2502). We also included in the model, as nuisance variables, the following RNA-seq metrics: average fragment length, exonic rate, number of genes detected in sample preparation method and read length.

### Phenotypes

*EPO, discovery phase*: We received the values of 5887 serum EPO level measurements of 4187 individuals from The National University Hospital of Iceland. The hospital laboratory estimated the EPO serum level with solid phase enzyme-labelled chemiluminiscent immunoassay using Immulite 1000 (Siemens Healthcare Diagnostics).

*EPO, replication phase (human erythropoietin immunoassay)*: Serum EPO concentration was measured by double-antibody sandwich ELISA (Human Erythropoietin Quantikine IVD ELISA kit #DP00; R&D Systems). The manufacturer’s protocol was followed according to instructions. Undiluted serum samples from 34 age-matched carrier and control pairs were applied in triplicate. The Shaker Method was used with 1 h of incubation periods followed by a total of 4 washes. Development in substrate solution was 25 min. Results were analysed using GloMax Discover System (Promega). The reported range for this assay is 2.5 200 mIU ml^−1^.

*Haemoglobin*: Haemoglobin concentration measurements of 273,160 Icelanders were obtained from four different laboratories in Iceland from 1993 to 2016. Of these, 137,064 were chip-typed using Illumina chips and their genotypes were imputed using long-range phased haplotypes. Genotype probabilities were computed for 136,096 individuals with chip-typed first- or second-degree relatives. Haemoglobin concentration measurements for each sex and the four different laboratories were separately transformed to a standard normal distribution and adjusted for age using a generalised additive model^51^.

### Code availability

We used publicly available software (URLs listed below) in conjunction with the above described algorithms in the sequencing processing pipeline (whole-genome sequencing, association testing, RNA-seq mapping and analysis): BWA 0.7.10 mem, https://github.com/lh3/bwa; GenomeAnalysisTKLite 2.3.9, https://github.com/broadgsa/gatk/; Picard tools 1.117, https://broadinstitute.github.io/picard/; SAMtools 1.3, http://samtools.github.io/; Bedtools v2.25.0-76-g5e7c696z, https://github.com/arq5x/bedtools2/; Variant Effect Predictor https://github.com/Ensembl/ensembl-vep. Variants were imputed based on the IMPUTE HMM model. We used R extensively to analyse data and create plots.

### Data availability

Sequence variants passing GATK filters have been deposited in the European Variation Archive, accession number PRJEB15197. RNA-seq data have been deposited in the Gene Expression Omnibus, accession number GSE102870.

## Electronic supplementary material

Supplementary Information
