# Supplementary material for: A truncating mutation in EPOR leads to hypo-responsiveness to erythropoietin with normal haemoglobin
Source: Commun Biol. 2018 May 17;1:49. doi: 10.1038/s42003-018-0053-3 (PMC6123817; doi:10.1038/s42003-018-0053-3)
Supplement: Supplementary file 1 — Supplementary Information [file 42003_2018_53_MOESM1_ESM.pdf]

Supplementary Table 1: Descriptive statistics of the population used in the GWAS of serum EPO levels.

	GWAS	Replication
**Individuals with EPO measure (N)**	4,187	68 (34 pairs)
**Mean age at measure (years)**	67.0	51.0
**Percent female (%)**	49.8	57.1
**Gln82Ter carriers (N)**	7	34
**Median EPO (Q1,Q3) (IU/L)**	13.3 (8.4, 22.7)	12.0 (6.9, 23.1)
**Non-carrier median EPO (Q1,Q3)**	13.2 (8.4, 31.8)	6.9 (5.5, 9.1)
**Carrier median EPO (Q1,Q3)**	70.6 (40.8, 2007.1)	22.3 (14.3, 32.8)

Supplementary Table 2: Most common diagnoses observed in the group of 4,187 individuals used in the GWAS discovery phase. The number of individuals from the GWAS discovery phase with the diagnosis is listed (N_{GWAS}), as well as the total size of the Icelandic phenotype list (N_{Total}).

Phenotype	N_{GWAS}	N_{Total}
Hypertension	2,492	54,974
Chronic kidney disease	2,391	25,608
Coronary artery disease	1,668	38,918
Chronic ischemic heart disease	1,405	26,455
Acute kidney injury	1,379	11,257
Heart failure	1,273	15,237
Atrial fibrillation and flutter	999	14,710
Alzheimer's disease	996	18,453
Elevated Hb levels*	1,400	25,544

*A total of 273,160 Icelanders had available haemoglobin measurements. Elevated haemoglobin levels were defined as at least one measurement over 171g/L for men, and 152g/L for women.

Supplementary Table 3: Association of rs370865377 with cardiovascular phenotypes commonly associated with elevated serum EPO levels.

Phenotype	# Cases	# Controls	P-value	OR	95% CI
**Ischemic Stroke**	5,626	262,087	0.98	0.99	0.45 - 2.17
**Myocardial Infarction**	23,965	311,807	0.38	1.22	0.78 - 1.90
**Venous Thromboembolism**	4,967	343,276	0.91	0.96	0.47 - 1.95

OR = odds ratio

Supplementary Table 4: Effects of rs7776054 in Iceland and in the Netherlands

Iceland N = 4,187			The Netherlands[17] N = 6,777			Meta-analysis				
P-value	Effect (SD)	95% CI	P-value	Effect (SD)	95% CI	P-value	Effect (SD)	95% CI	I^2	P-het
2.6×10^{-5}	0.10	0.05-0.15	8.8×10^{-21}	0.29	0.23-0.35	1.8×10^{-19}	0.17	0.13-0.21	95.8	1.2×10^{-6}

CI = confidence interval. I^2 = meta-analysis heterogeneity. P-het = P value of heterogeneity

Supplementary Table 5: Comparison of effects of the Icelandic EPOR N-terminal truncating mutation and the Finnish C-terminal truncating mutation on serum EPO levels, RBC count, haemoglobin levels, and EPO-R responsiveness to EPO.

	Iceland	Finland[16]
**EPO**	↑	↓
**RBC**	↔	↑
**Haemoglobin**	↔	↑
**EPO-R responsiveness**	↓	↑

↑ = increase, ↓ = decrease, ↔ = unchanged

Supplementary Table 6: Summary of genotyping chips used to genotype 151,677 Icelanders. Some individuals have been genotyped using more than one unique chip.

Chip name	Chip description	Nr. of SNPs	Chip-typed (N)	Individuals used in GWAS discovery (N)
OmniExpress	HumanOmniExpress	725,095	49,482	698
OmniExpress24	HumanOmniExpress-24	714,758	33,894	238
HumanHap 300	Illumina HumanHap 317 K SNP Chip	317,870	22,429	943
HumanHapCNV370	Merge of HumanHap300 and Human CNV chips	371,900	14,138	469
Human Omni1	1 M SNP Chip redesigned, 500 K diff versus normal 1 M	1,137,466	10,859	255
OmniExpPlus	DECODE	706,534	9,725	3
Omni2.5-8	HumanOmni2.5-8	2,379,855	3,948	220
OmniExpMulti	HumanOmniExp-12v1MultiUse	730,525	2,808	77
HumanOmni2.5	HumanOmni2.5	2,443,177	2,352	53
HumanHap 1 M	Illumina HumanHap 1 M SNP Chip	1,136,004	1,267	29
Omni5	HumanOmni5-4v1	4,301,332	661	5
HumanHap 610	Illumina Human610-Quad v1 SNP Chip	620,901	646	11
Omni2.5Multi	HumanOmni2.5-4v1-Multi_D	2,443,177	400	21
Human660W	Human660W	655,214	22	0

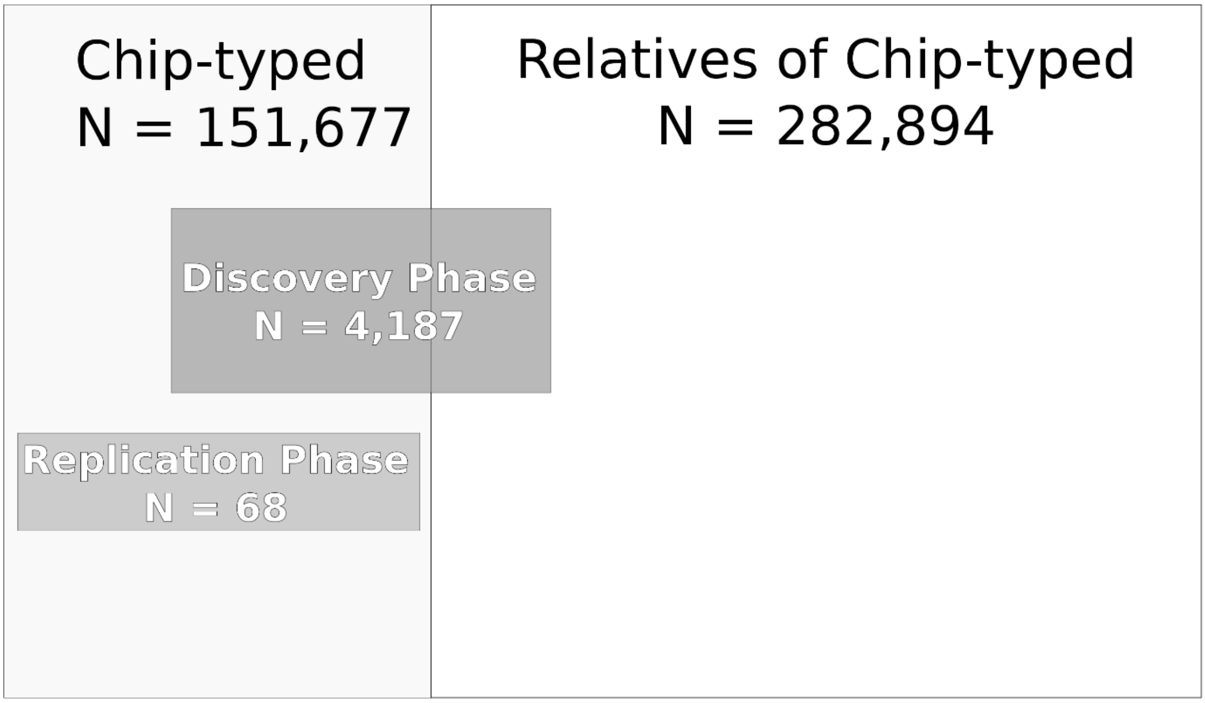

Supplementary Figure 1: Schematic of samples used in the GWAS discovery phase and replication phase within the Icelandic population. 2,994 individuals used in the GWAS discovery phase were chip-typed and 1,193 individuals were first- and second degree relatives of chip-typed individuals and had erythropoietin levels measured in the clinical context. There was no overlap between the discovery and replication phase groups. The 68 individuals used in the replication phase consisted of 34 Gln82Ter carriers and 34 matched controls.

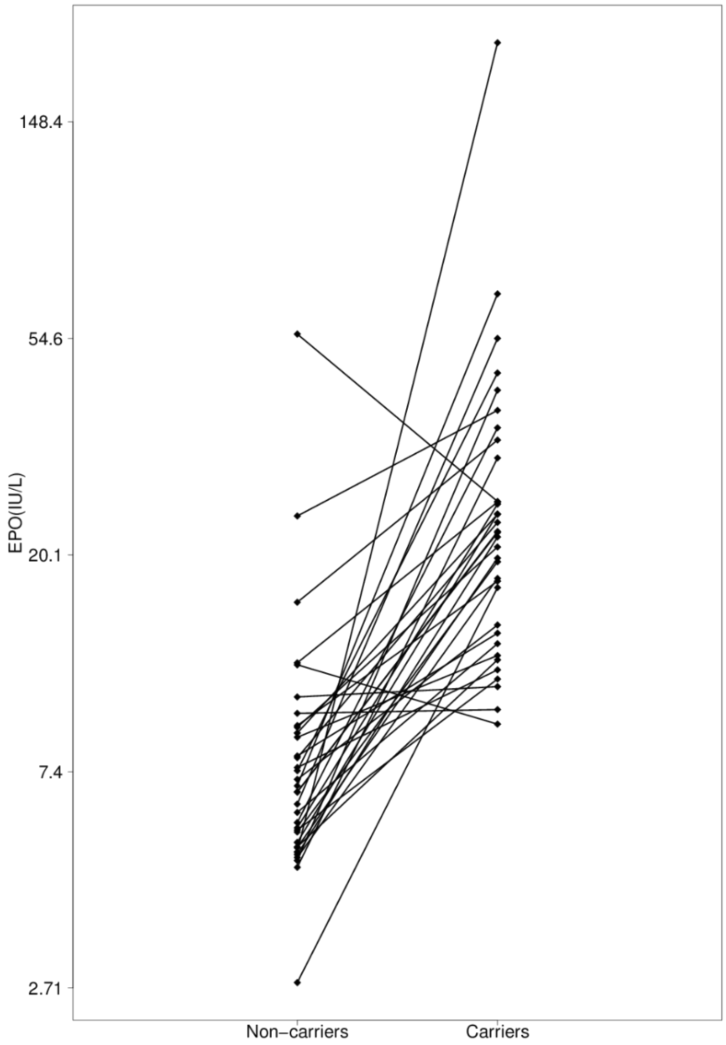

Supplementary Figure 2: Serum Erythropoietin level for carriers of rs370865377[A] and matching non-carriers. Serum Erythropoietin (EPO) levels (IU/L) are log-transformed (y-axis) for carriers of rs370865377[A] and matching controls (non-carriers). Carriers and non-carriers were matched based on sex, sampling date and age at sampling. Matched case-control pairs are represented with a line connecting them. Serum EPO levels were significantly higher for carriers than controls (P = 1.7x10-6). Only 3 out of 34 carriers had lower EPO serum level than their matching control.

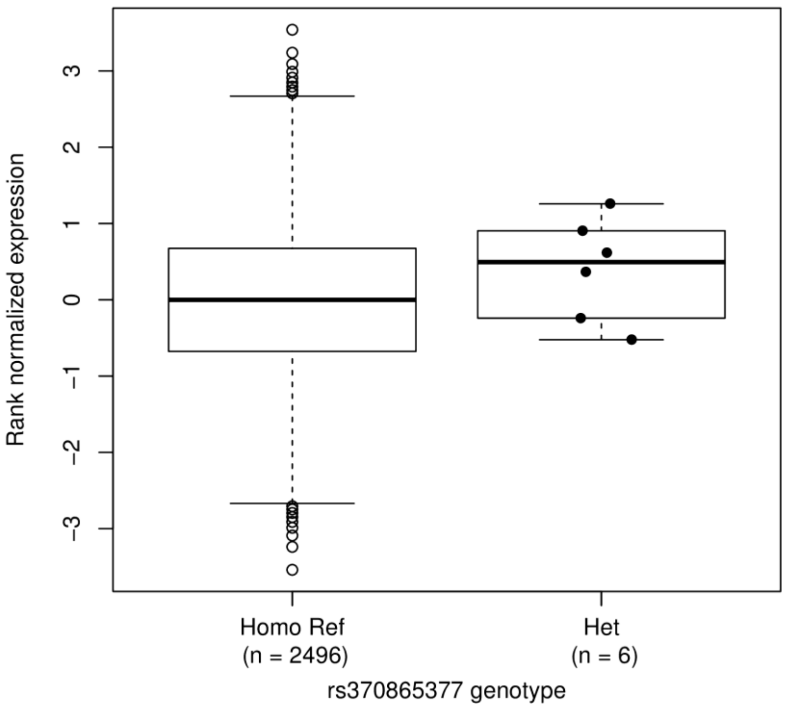

Supplementary Figure 3: Rank-normalized expression levels of *EPOR* mRNA from whole-blood samples of 2,502 Icelanders stratified by rs370865377[A] genotype (Effect = 0.3 SD, P = 0.48). The bottom and top of each box represent the first and third quantiles, the line inside the box is the median and whiskers represent the ±1.5 times the interquartile range.

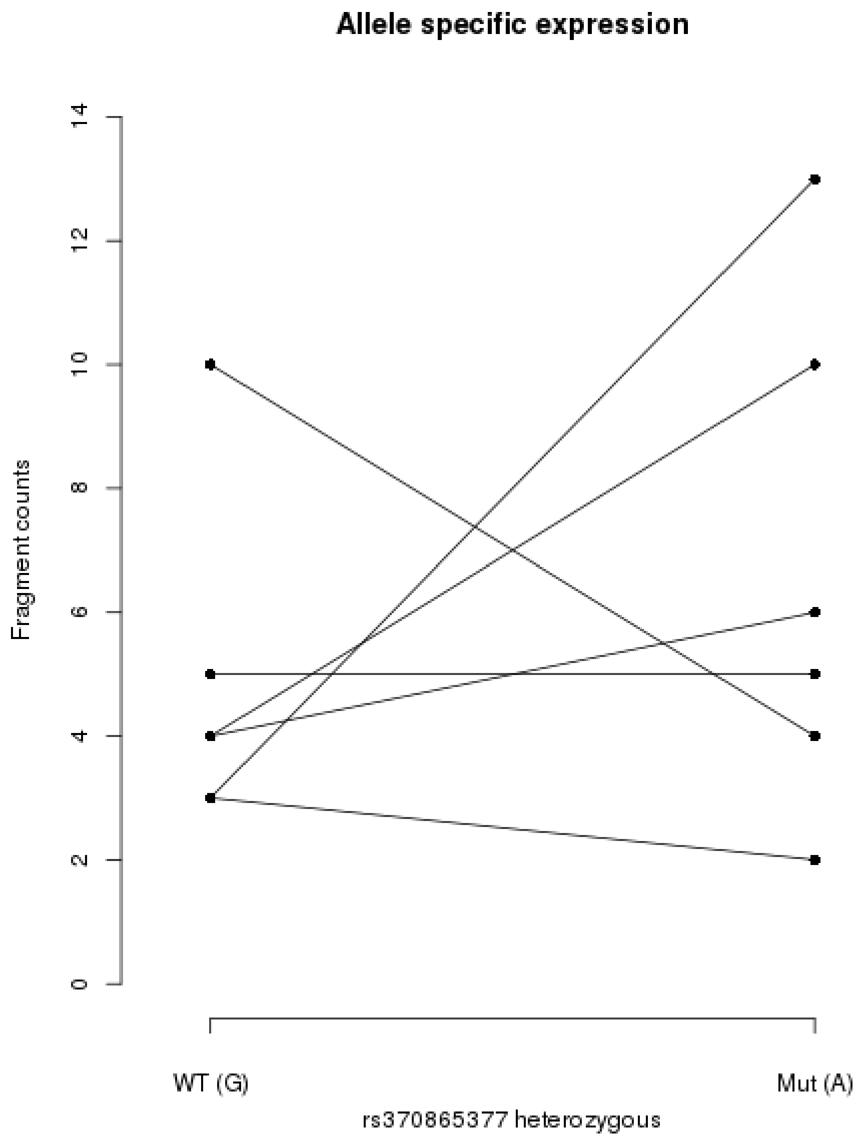

Supplementary Figure 4: Allele-specific expression of *EPOR* mRNA fragments from whole-blood samples of 6 heterozygous rs370865377[A] carriers. Matched allele-specific fragment counts per individual are connected by a black line. Expression is not significantly different between the wild-type (WT) and mutated (Mut) alleles (P = 0.46).

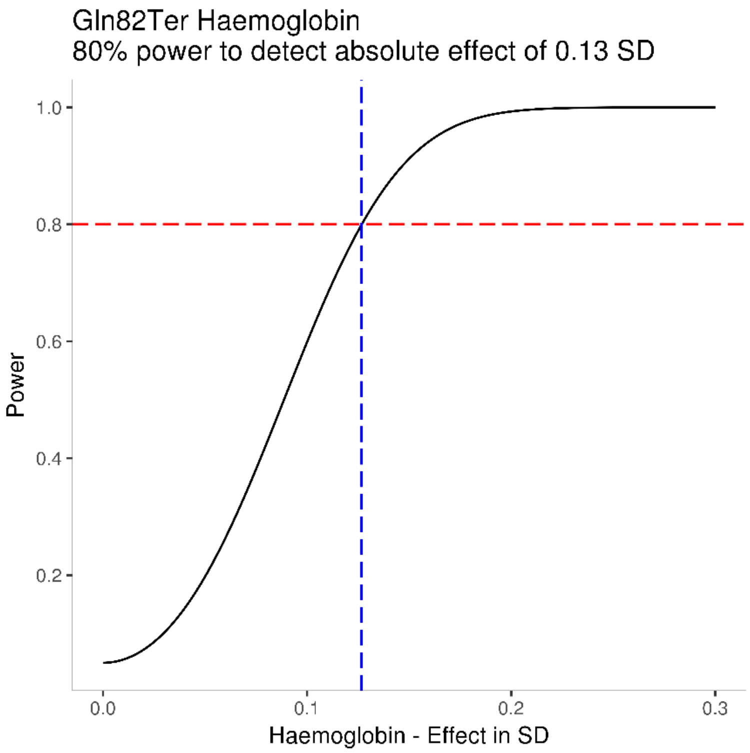

Supplementary Figure 5: Estimation of power to detect association of Gln82Ter with serum haemoglobin levels for all possible effect sizes given the standard error. At the two sided significance level of 0.05 we had 80% power to detect an absolute effect of 0.127 SD (corresponding to 2.03 g/L or 1.5% of mean) for Gln82Ter on haemoglobin levels.
